# Supplementary material for: Synaptic ribbon dynamics after noise exposure in the hearing cochlea
Source: Commun Biol. 2024 Apr 6;7:421. doi: 10.1038/s42003-024-06067-y (PMC10998851; doi:10.1038/s42003-024-06067-y)
Supplement: Supplementary file 2 — Supplementary Information [file 42003_2024_6067_MOESM2_ESM.pdf]

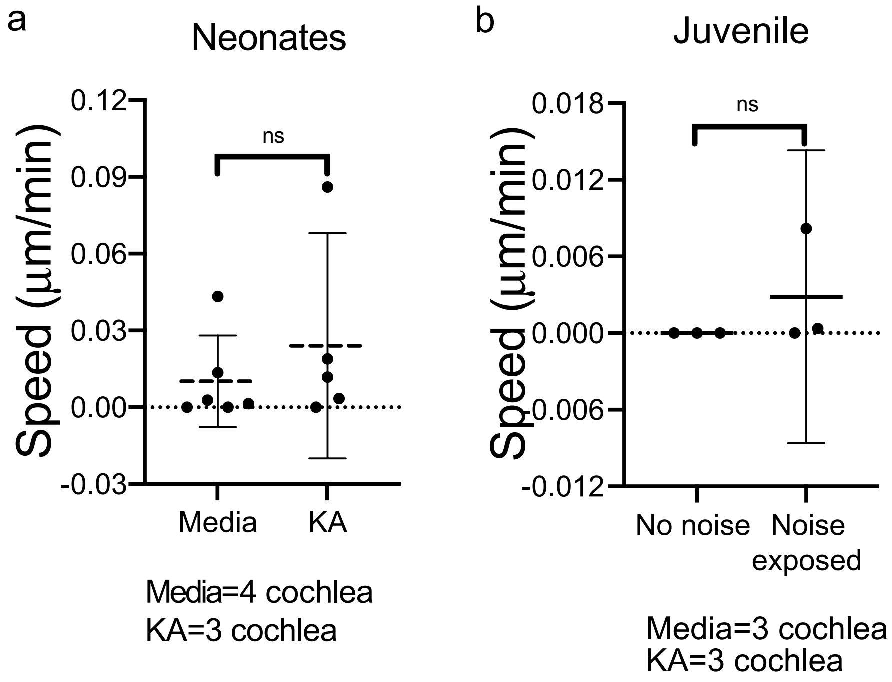

Figure S1. Stability of FM1-43 reference frame. Speed of the hair-cell reference frame comprising hair cells labelled with FM1-43 was measured in (a) Media vs KA treated neonate cochlea and (b) no-noise or noise-exposed juvenile cochlea. ns= not significant.

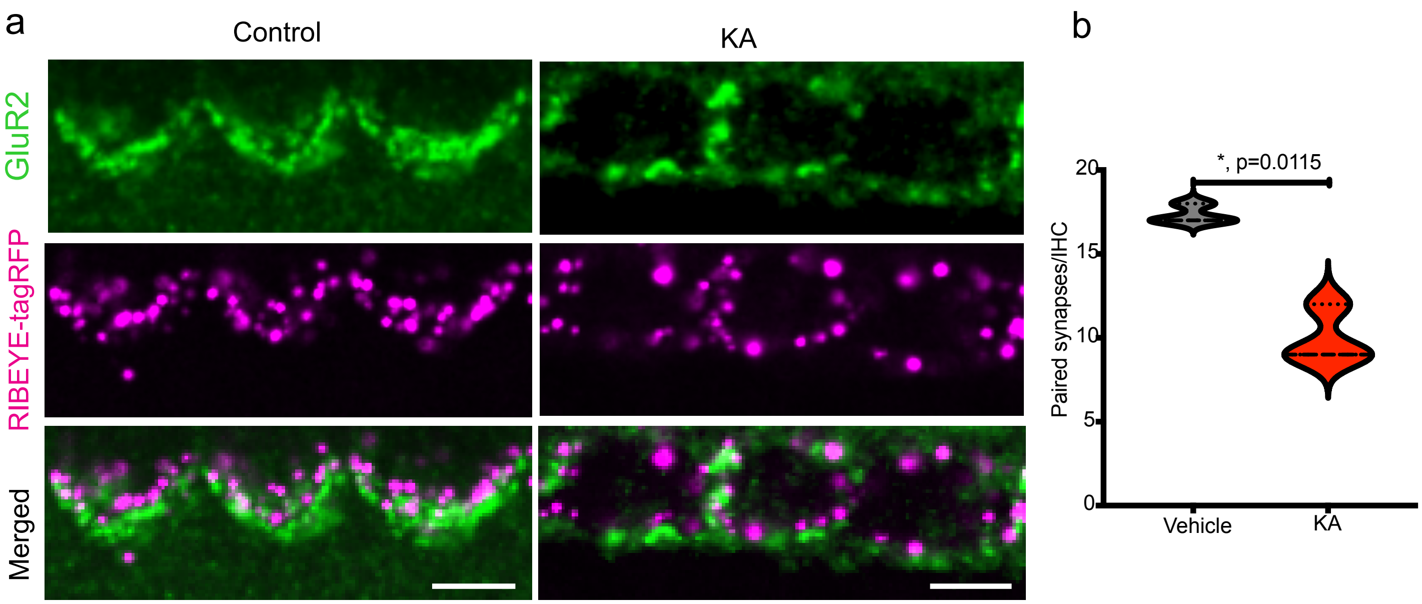

Figure S2. Loss of paired synapses after excitotoxic lesioning. a. Identification of paired synapses in fixed neonatal cochlea treated with KA as a model for glutamate excitotoxicity. Scale bars = 5 µm. b. Quantification of paired synapses 24 hr after 2 hr treatment with KA vs vehicle (control). N = 15 (vehicle) and 12 (KA) IHCs from 3 cochleae for each condition. *, $p < 0.05$.

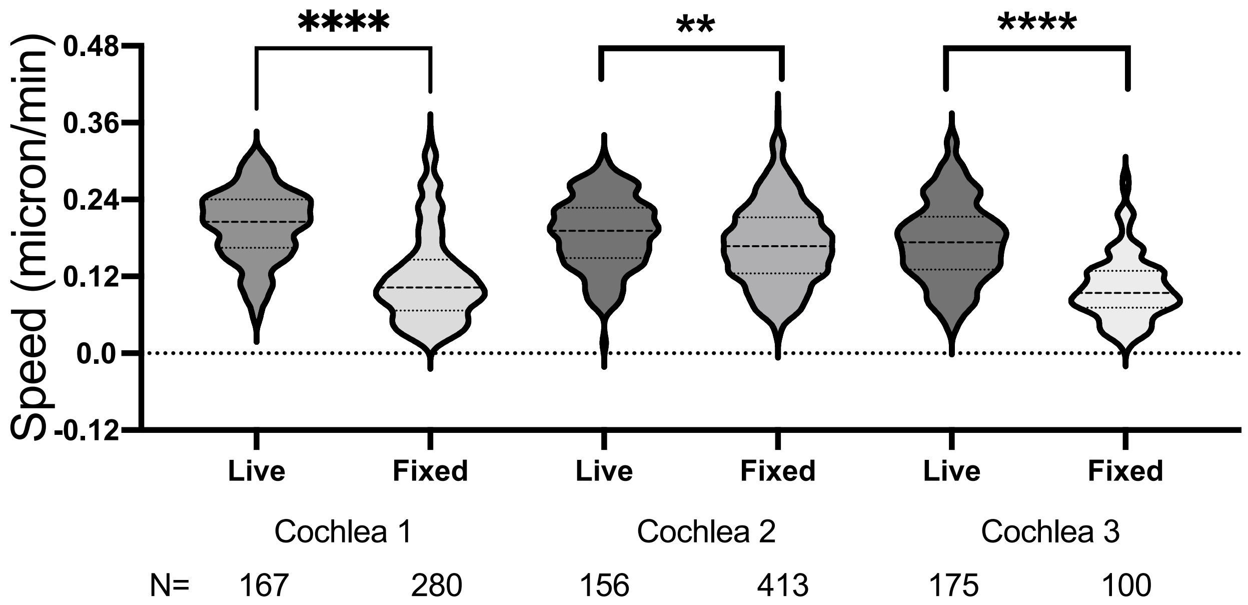

Figure S3. Punctal speed in fixed versus live cochlea. We measured the speed of RIBEYE-tagRFP puncta in 3 neonatal cochleae which measured using live imaging and after fixation. N=number of puncta. ****, $p<0.0001$, **, $p=0.0027$.

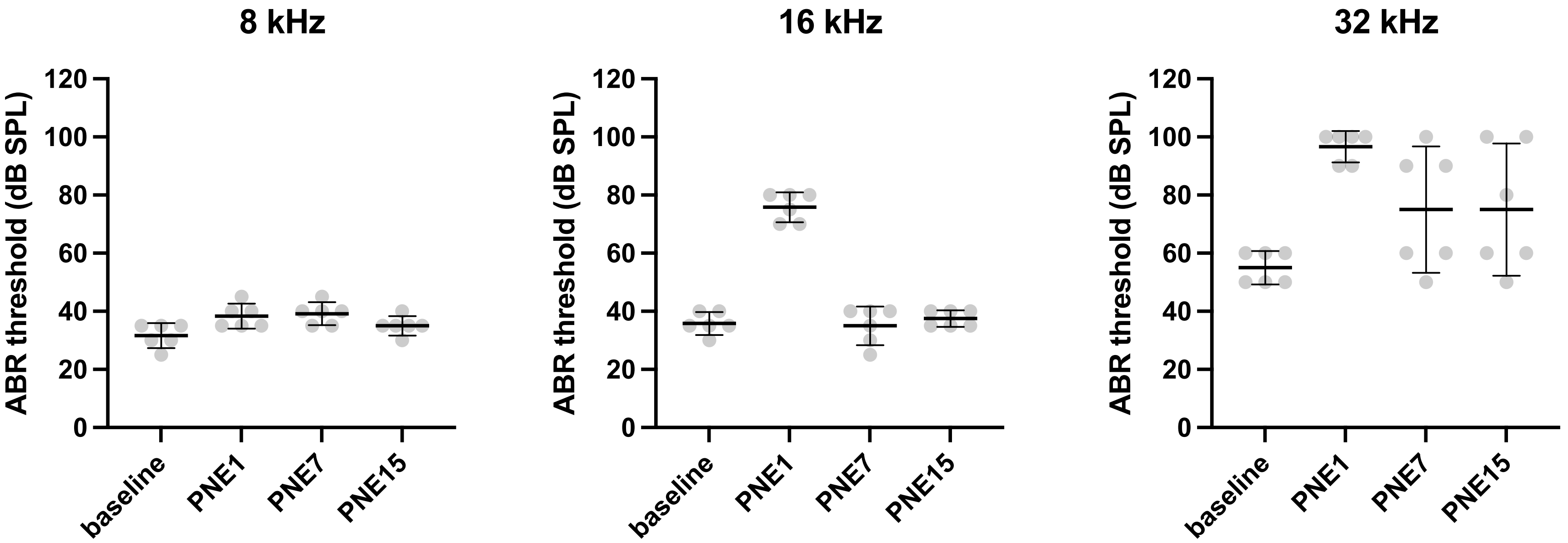

Figure S4. Noise-induced hearing loss and synaptopathy in RIBEYE-tagRFP mice. A. Auditory brainstem response (ABR) thresholds were measured in male (N=3)_and female (N=3) RIBEYE-tagRFP mice at 8, 16, and 32 kHz, demonstrating elevation at post-noise-exposure (PNE) days 1 and 7, with complete recovery by PNE14 at 8 and 16 kHz and partial recovery at 32 kHz.

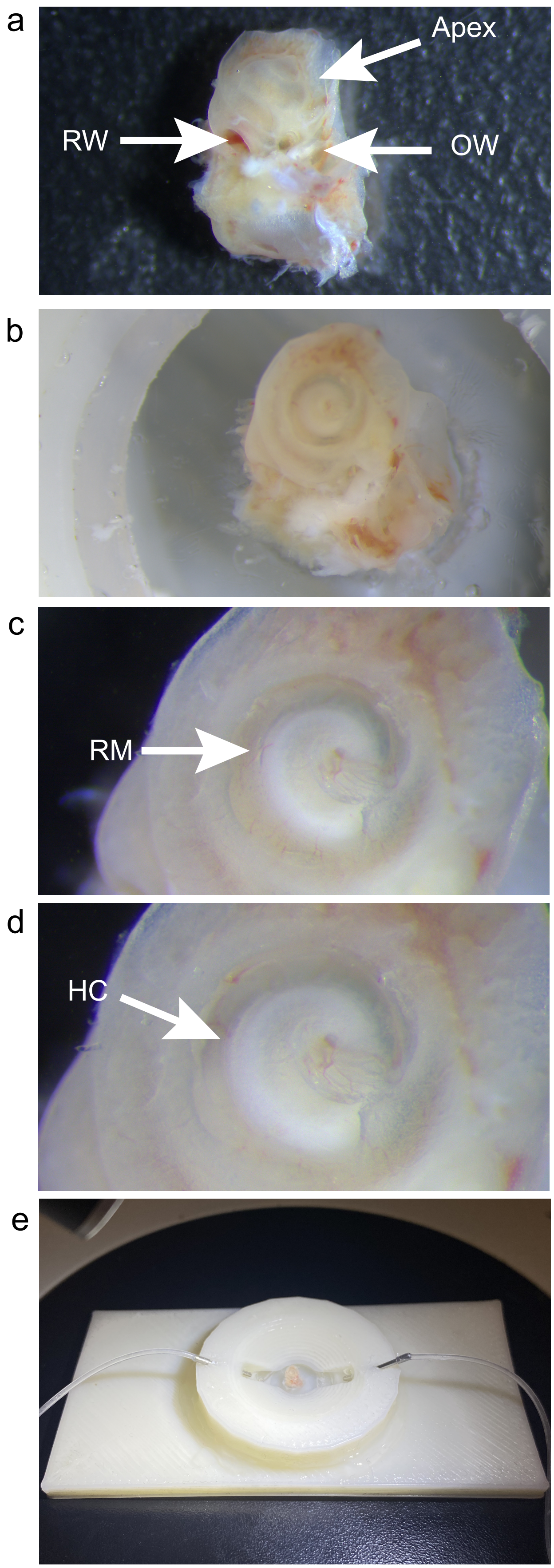

Figure S5. Preparation of juvenile, mature-hearing cochlea for live imaging.
a. The otic capsule is grossly extracted from the temporal bone utilizaing suture lines around the cochlea. RW: round window (RW); OW: oval window. b. The temporal bone is then snapped into place into a contained in the temporal bone placed into a hole made in a plastic coverslip with the apex facing directly upward. c. The bone overlying the helicotrema and cochlear apex is flicked off with a #11 scalpel. RM: Reissner's membrane. d. Reissner's membrane is removed, revealing the rows of hair cells (HC) for imaging. e. Overview of the temporal bone mounted on the custom-designed 3D slide.

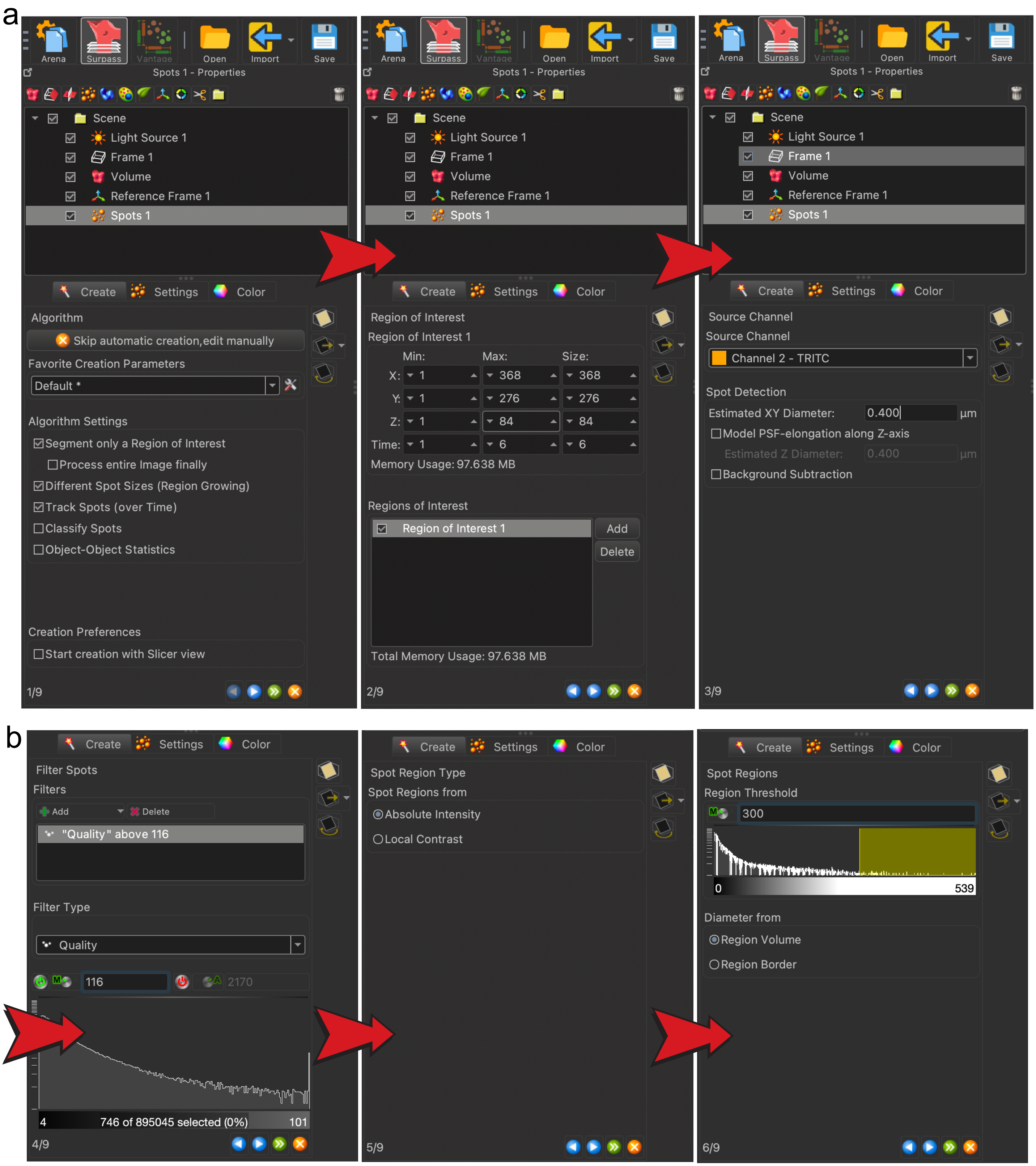
a
Arena
Surpass
Vantage
Open
Import
Save
Spots 1 - Properties
Scene
Light Source 1
Frame 1
Volume
Reference Frame 1
Spots 1
Create
Settings
Color
Algorithm
Skip automatic creation,edit manually
Favorite Creation Parameters
Default *
Algorithm Settings
Segment only a Region of Interest
Process entire Image finally
Different Spot Sizes (Region Growing)
Track Spots (over Time)
Classify Spots
Object-Object Statistics
Creation Preferences
Start creation with Slicer view
1/9
Region of Interest
Region of Interest 1
Min:
Max:
Size:
X: 1 368 368
Y: 1 276 276
Z: 1 84 84
Time: 1 6 6
Memory Usage: 97.638 MB
Regions of Interest
Region of Interest 1
Add
Delete
Total Memory Usage: 97.638 MB
2/9
Source Channel
Source Channel
Channel 2 - TRITC
Spot Detection
Estimated XY Diameter: 0.400 µm
Model PSF-elongation along Z-axis
Estimated Z Diameter: 0.400 µm
Background Subtraction
3/9
b
Filter Spots
Filters
Add
Delete
"Quality" above 116
Filter Type
Quality
116
2170
4
746 of 895045 selected (0%)
101
4/9
Spot Region Type
Spot Regions from
Absolute Intensity
Local Contrast
5/9
Spot Regions
Region Threshold
300
0
539
Diameter from
Region Volume
Region Border
6/9

C

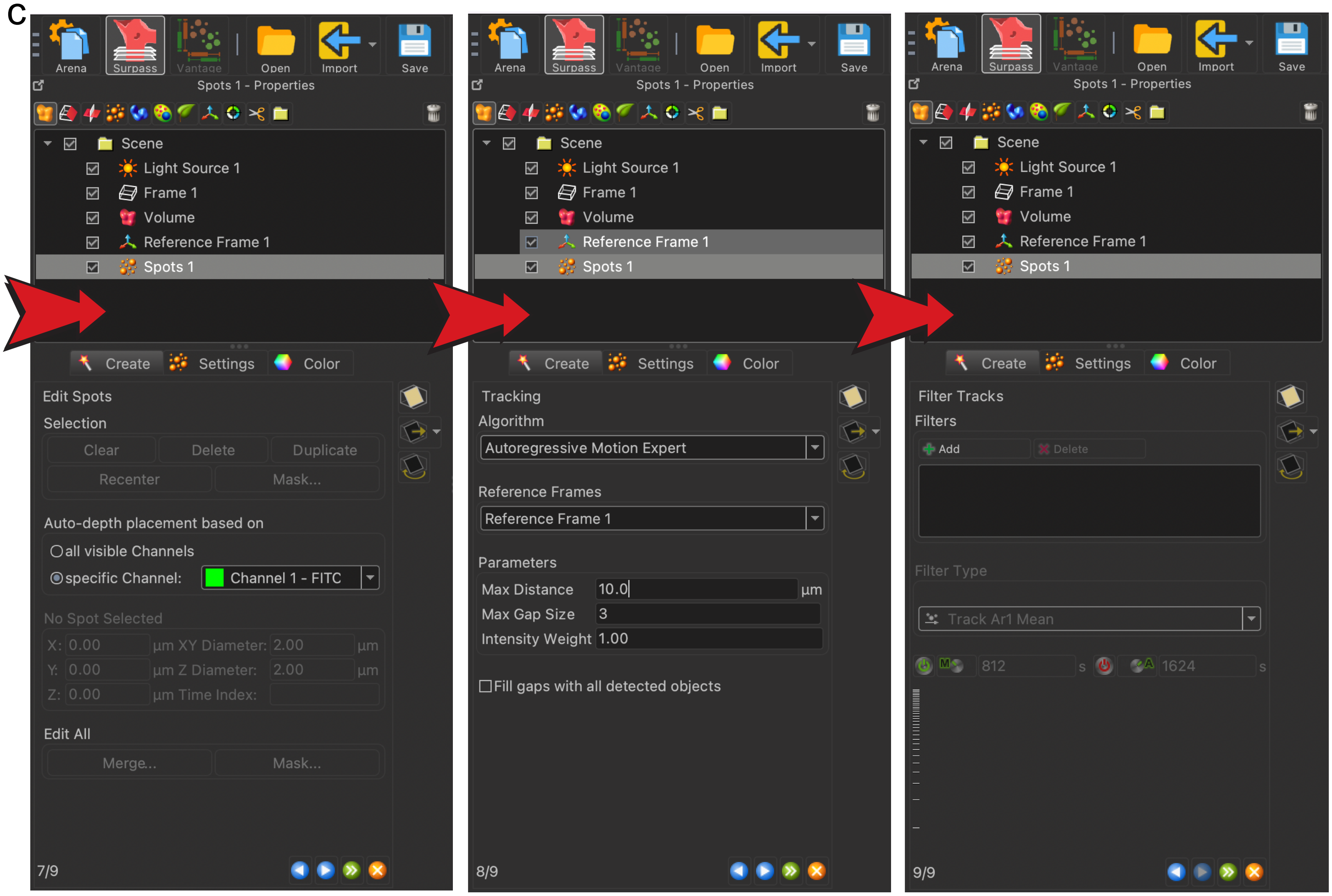

Figure S6. Image processing to identify and track pre-synaptic puncta. Walkthrough in detail of the steps for RIBEYE-tagRFP puncta selection, rendering, and tracking in Imaris. a. Three-dimensional confocal micrographs were converted to .ims format using ImarisFileConverter 9.9.1, then opened in Imaris (v9.9.1). The Spots function was used to create 3D-rendered puncta after manual segmentation of a volumetric Region of Interest. Different spot sizes (growing) and Track Spots (over time) were selected. Region of interest was specified, followed by selecting the source channel (TRITC). Estimated XY diameter was set to 0.4 µm for spot detection. b. Spots were filtered by designating a Quality threshold (in this case, 116, for neonatal images). Spot regions were selected to be selected from Absolute intensity, and the Region Threshold was set at 300. c. Auto-depth placement was performed using the FITC channel, followed by selection of Autoregressive Motion Expert as a tracking algorithm. The previously created Reference Frame 1 was selected as the Reference Frame. Max Distance, Max Gap Size, and Intensity Weight were set to 10 µm, 3 µm and 1 µm, respectively. Next, the creation wizard was finished and terminated. Red arrows: successive steps.
